# Hydroxychloroquine (HCQ) Modulates Autophagy and Oxidative DNA Damage Stress in Hepatocellular Carcinoma to Overcome Sorafenib Resistance via TLR9/SOD1/hsa-miR-30a-5p/Beclin-1 Axis

**DOI:** 10.3390/cancers13133227

**Published:** 2021-06-28

**Authors:** Ming-Yao Chen, Vijesh Kumar Yadav, Yi Cheng Chu, Jiann Ruey Ong, Ting-Yi Huang, Kwai-Fong Lee, Kuen-Haur Lee, Chi-Tai Yeh, Wei-Hwa Lee

**Affiliations:** 1 Division of Gastroenterology and Hepatology, Department of Internal Medicine, School of Medicine, College of Medicine, Taipei Medical University, Taipei 110, Taiwan; 08350@s.tmu.edu.tw (M.-Y.C.); 20604@s.tmu.edu.tw (V.K.Y.); 2 Division of Gastroenterology and Hepatology, Department of Internal Medicine, Shuang Ho Hospital, New Taipei City 23561, Taiwan; 3 Department of Medicine, St. George’s University School of Medicine, St. George SW17 0RE, Grenada; ychu@sgu.edu; 4 Department of Emergency Medicine, Taipei Medical University-Shuang Ho Hospital, New Taipei City 23516, Taiwan; malsia95@gmail.com; 5 Department of Emergency Medicine, School of Medicine, Taipei Medical University, Taipei 110, Taiwan; 6 Biobank management Center, Taipei Medical University-Shuang Ho Hospital, New Taipei City 23561, Taiwan; 15729@s.tmu.edu.tw (T.-Y.H.); 19118@s.tmu.edu.tw (K.-F.L.); 7 Graduate Institute of Cancer Biology and Drug Discovery, College of Medical Science and Technology, Taipei Medical University, Taipei 110, Taiwan; 8 Cancer Center, Wan Fang Hospital, Taipei Medical University, Taipei 110, Taiwan; 9 Department of Medical Research & Education, Taipei Medical University Shuang Ho Hospital, New Taipei City 23561, Taiwan; 10 Department of Medical Laboratory Science and Biotechnology, Yuanpei University of Medical Technology, Hsinchu 300, Taiwan; 11 Department of Pathology, Taipei Medical University Shuang Ho Hospital, New Taipei City 23561, Taiwan; whlpath97616@s.tmu.edu.tw

**Keywords:** hepatocellular carcinoma, TLR9, HCQ, autophagy, oxidative stress, sorafenib resistance

## Abstract

**Simple Summary:**

Hepatocellular carcinoma (HCC) is one of the major causes of cancer-associated death worldwide. Development of sorafenib resistance presents a major failure of HCC therapy. We applied a combination therapy approach of hydroxychloroquine (HCQ)–sorafenib, both in vitro and in vivo, with data demonstrating the synergistic effect of HCQ in modulating the expression of toll-like receptor (TLR)-9 and regulating the cancer cells stemness, mesenchymal state, autophagy, and sorafenib resistance through inducing the antioxidant superoxide dismutase (SOD)-1 and apoptosis-allied gene expression and reducing the oxidative DNA damage stress in HCC sorafenib-resistant cells via hsa-miR-30a-5p epigenetic regulation axis. Thus, the combination of HCQ–sorafenib provides a suitable approach in improving therapeutic outcomes of sorafenib-resistant HCC patients.

**Abstract:**

Sorafenib is used for treating advanced hepatocellular carcinoma (HCC), but some patients acquire sorafenib resistance. We investigated the mechanisms underlying acquired sorafenib resistance in HCC cells and targeted them to re-sensitize them to sorafenib. In silico analysis indicated that toll-like receptor (TLR)-9 was significantly overexpressed, and that miRNA (hsa-miR-30a-5p) was downregulated in sorafenib-resistant HCC cells, which modulated HCC cell proliferation, oxidative stress, and apoptosis. TLR9 overexpression increased HCC cell proliferation, whereas TLR9 inhibition from hydroxychloroquine (HCQ) decreased HCC cell proliferation, tumor growth, oxidative stress marker (SOD1), and the formation of autophagosome bodies (reduced ATG5 and Beclin-1 expression). Moreover, HCQ treatment reduced epithelial–mesenchymal transition, leading to decreased clonogenicity, migratory ability, and invasiveness. HCQ targeted and reduced the self-renewal capacity phenotype by inhibiting tumorsphere generation. Both in vitro and in vivo results demonstrated the synergistic effect of the HCQ–sorafenib combination on sorafenib-resistant HCC (Huh7-SR) cells, increasing their sensitivity to treatment by modulating TLR9, autophagy (ATG5 and Beclin-1), oxidative stress (SOD1), and apoptosis (c-caspase3) expression and thus overcoming the drug resistance. This study’s findings indicate that TLR9 overexpression occurs in sorafenib-resistant HCC cells and that its downregulation aids HCC suppression. Moreover, HCQ treatment significantly increases sorafenib’s effect on sorafenib-resistant HCC cells.

## 1. Introduction

Hepatocellular carcinoma (HCC) is the fifth most common type of liver cancer [1]. It is a major medical burden and is associated with fatal malignancy and cancer-related deaths worldwide [2,3]. The 5-year survival rate in patients with HCC is less than 80% [4]. Many external and environmental factors, such as viral infection and drug abuse, contribute to rapid liver damage and eventual progress to HCC. Liver resection and, eventually, transplantation are the more effective and adopted curative measures of HCC [5].

Sorafenib remains the most extensively and effectively used chemotherapeutic agent in advanced-staged HCC [5,6]. However, its effect is short-lived because patients with HCC who initially respond very well to sorafenib can develop chemoresistance [7]. Genomic and transcriptional instabilities have been observed in patients with advanced-stage HCC, causing HCC treatment failure and sorafenib-targeting mutation and resistance [8]. Moreover, oxidative stress plays a pivotal role in HCC development, progression, and chemoresistance [9,10].

A low level of reactive oxygen species (ROS) is indispensable in the physiological processes of cell proliferation, viability, apoptosis, cell cycle arrest, and senescence [11]. By contrast, increased cellular ROS levels increase oxidative stress and create a potentially toxic environment, disrupting the balance between ROS production and oxidative defenses [12]. Oxidative stress contributes to cancer and drug resistance by enhancing DNA damage [13]. The free oxygen radicals (O_2_**^•^**– and H_2_O_2_) generated in this process are neutralized by enzymes such as superoxide dismutase (SOD) and catalase (CAT), thus protecting the cells from them [12]. The SOD family has three distinct members: SOD1 or copper/zinc SOD, SOD2 or manganese SOD, and SOD3 or extracellular SOD [12,14,15]. SOD1 is the major type of SOD in the cytoplasm, nucleus, and mitochondrial intermembrane space [12,15]. Because of its crucial role in mitochondrial function, its association with tumorigenesis is unlikely [16]. The functional effect of SOD1 remains controversial with specific regard to whether SOD1 overexpression prevents or induces tumor progression, especially in sorafenib-resistant HCC. Mutations in the SOD1 gene have been linked to numerous human diseases and cancers [17,18,19]. However, the role of SOD1 in oxidative DNA damage associated with HCC progression and chemoresistance remains underexplored. Moreover, the rapid development of chemoresistance impairs the success of cancer therapy.

Many studies have suggested that chronic inflammation, hepatic fibrosis, and immune activation are associated with the translocation of gut-harboring bacteria [20]. Notably, toll-like receptors (TLRs), specifically TLR9 and TLR7, are overexpressed in HCC [21]. In HCC, TLR9 stimulates proliferation and markedly reduces apoptosis, both in vivo and in vivo [21].

Micro-RNAs (miRNAs) are small (approximately 20 bp), endogenous, noncoding RNAs that play a key role in controlling many vital biological processes [22,23,24]. They target specific mRNAs and modulate their expression by degrading them and thus inhibit protein translation [25]. Some miRNAs, such as miR-222, have been associated with sorafenib resistance and cancer chemosensitivity [26,27]. Because of their many roles, these miRNAs have been attractive targets in research on early biomarker discovery.

Autophagy modulates the growth of cancer cells depending on the cell status [28]. Such modulation is an evolutionary conserved catabolic process in eukaryotic cells in which damaged or useless organelles are degraded and recycled by lysosomal systems [29,30,31]. The function of autophagy is more than homeostasis maintenance; it also plays a role in cancer [32] by acting as a factor in both tumor progression and tumor suppression [33]. Under cellular stress, such as nutrient deficiency, radiation, and chemotherapy, autophagy is activated to promote tumor cell survival and induce chemoresistance. Sorafenib induces autophagy and apoptosis, which may lead to sorafenib resistance in HCC [28].

HCQ is a well-known antimalarial agent. It is a key inhibitor of TLR9 and is used in the treatment of cancer, especially for its anti-HCC effect [34], often combined with conventional anticancer drugs [34,35]. HCQ targets both cancer cells and the tumor microenvironment (TME) [36]. It inhibits autophagosome–lysosome fusion (LC3 cell marker, ATG5 [28], and Beclin-1 [28] expressions) and reduces chemoresistance [37]. Another mechanism through which cancer cells, including HCC, avoid or resist chemotherapy is the development of cancer stem cells (CSCs), which can survive and withstand most of the chemotherapeutic agents that kill bulk tumors [38,39,40,41].

In this study, we explored the importance of TLR9 and how HCQ affects TLR9 expression. Our findings indicates that HCQ together with miRNA (hsa-miR-30a-5p) targets and re-sensitizes sorafenib-resistant HCC cells to sorafenib by modulating the expression of autophagosome formation, oxidative stress, CSCs, and oncogenic markers (epithelial–mesenchymal transition (EMT)) in HCC cells both in vitro and in vivo. In brief, our findings indicate the pivotal role of HCQ in re-sensitizing sorafenib-resistant HCC cells to sorafenib by impeding autophagy and oxidative DNA damage stress via the TLR9/SOD1/hsa-miR-30a-5p/Beclin-1 axis.

## 2. Materials and Methods

### 2.1. Cell Lines and Media

Human HCC, Huh7, and HepG_2_ HCC cell lines were purchased from the American Tissue Culture Collection. The cells were maintained under the conditions recommended by the vendor, cultured in Roswell Park Memorial Institute (RPMI) 1640 medium (Thermo Fisher Scientific, San Jose city, CA, USA), and supplemented with 10% fetal bovine serum (FBS) and 1% penicillin–streptomycin (Invitrogen, Portland City, OR, USA) at 37 °C and 5% humidified CO_2_.

### 2.2. Microarray Preprocessing and Analysis

The gene expression profile GSE94550 was downloaded from the Gene Expression Omnibus (GEO) database (www.ncbi.nlm.nih.gov/geo/, accessed on 20 July 2020), and the data of liver cancer patients (LIHC) RNA-seq expression results were downloaded from The Cancer Genome Atlas (TCGA) portal Xena browser (http://www.xenabrowser.net/, accessed on 10 July 2020) and used for survival analysis.

### 2.3. Differential Expression Analysis

To determine genes that were differentially expressed between sorafenib-resistant and sorafenib-sensitive (Huh7-SR vs. Huh7) cells in the GSE94550 database (www.ncbi.nlm.nih.gov/geo/, accessed on 20 July 2020), we employed the Bayes method and linear model using the R package *limma*. All *p*-values were adjusted using the false discovery rate controlling procedure of Benjamini–Hochberg. Genes with a log_2_-fold change of >1 and adjusted *p*-values of < 0.05 were considered significant.

### 2.4. Immunohistochemistry (IHC) Analyses

This study enrolled patients with HCC from the Taipei Medical University Shuang-Ho Hospital, Taipei, Taiwan. The study was reviewed and approved by the hospital’s institutional review board (TMU-JIRB: 201302016). Collected samples were fixed in 4% paraformaldehyde and embedded in paraffin, with 5 μm thick sections cut from the paraffin blocks. Staining was performed using anti-TLR9 (1:5000; cat. no. ab37154, Abcam), followed by secondary antibody and hematoxylin and eosin (H&E).

### 2.5. Cell Viability Test and Calculation of the Combination Index

Stocks of HCQ and sorafenib were prepared by dissolving 20 mg/mL in DMSO and stored at −20 °C until use. The effects of HCQ and sorafenib on cell proliferation were detected by the sulforhodamine B (SRB) assay. The synergistic effect of and interaction between the two drugs was analyzed using isobolograms of the drug combination and the median-effect principle, respectively, as reported by Chou and Talalay [42,43]. The combination index (CI) was calculated using CompuSyn software. CI < 1 represented synergism [43]. Briefly, the HCC cells were seeded in 96-well plates (3 × 10^3^ cells/well) and treated with drugs (HCQ, sorafenib, or both) at the specified concentrations and times. After the respective drug treatments, the relative cell number was estimated by applying SRB reagent according to the manufacturer’s protocol (Sigma, Vallejo City, CA, USA).

### 2.6. Apoptosis and Cell Cycle Analysis

Apoptosis and cell cycle were analyzed using flow cytometry (Beckman, Fullerton, CA, USA) with an annexin V/7AAD (FITC-conjugated) apoptosis kit (Biolegend, Foster City, CA, USA) or propidium iodide (PI), according to the manufacturer’s protocol. To determine the effect on the cell cycle, we exposed HCC cells to HCQ and sorafenib alone or in combination for 48 h. Thereafter, cells were washed and fixed with 70% ethanol. HCC cells were washed, resuspended, and stained with PI 10 µg/mL in PBS for 30 min at room temperature in the dark. Cells were analyzed with flow cytometry (Becton-Dickinson, Mountain View, CA, USA), and the population of cells in each phase was calculated.

### 2.7. Western Blotting and qRT-PCR

Cells were washed with PBS and then lysed in RIPA lysis buffer. Cellular protein lysates were isolated using a Protein Extraction Kit (QIAGEN, Portland City, OR, USA) and quantified by a Bradford Protein Assay Kit (Promega, Foster city, CA, USA). A total of 20 μg of samples from different experiments were loaded and subjected to run on SDS-PAGE using a Mini-Protean III system (Bio-Rad, Taipei City, Taiwan). Separated proteins were transferred onto a polyvinylidene fluoride (PVDF) membrane using a Trans-Blot Turbo Transfer System (Bio-Rad, Taipei City, Taiwan), followed by blocking with Tris-buffered saline plus skim milk. These PVDF membranes were then probed with respective primary antibodies, followed by secondary antibodies. An ECL detection kit was used for the detection of the protein of interests. Images were captured and analyzed using a UVP BioDoc-It system (Upland, Durham, NC, USA). qRT-PCR was performed by isolating total RNA per a TRIzol-based protocol (Life Technologies, Los Angeles City, CA, USA), according to the manufacturer’s instructions. Two micrograms of total RNA were reverse-transcribed using a QIAGEN OneStep RT-PCR Kit (QIAGEN, Portland City, OR, USA), and the PCR reaction was performed using a Rotor-Gene SYBR Green PCR Kit (QIAGEN, Portland City, OR, USA). The primary antibody details are shown in Table S1 for genes used in this study.

### 2.8. Colony Formation Assay

A colony-forming assay was performed per the method of a previous study [44], with slight modifications. Briefly, 300 colon cancer cells were seeded in six-well plates and treated with HCQ and sorafenib alone or in combination. The cells were allowed to grow for another week, and they were then harvested, fixed, and counted.

### 2.9. Wound Healing Migration Assay

Cells were seeded in six-well plates (Corning, Corning, NY, USA) with RPMI 1640 medium containing 10% FBS and cultured to 95–100% confluence. A scratch along the median axis was made with a sterile yellow pipette tip across the cells. Cell migration pictures were captured immediately and at 48 h after the scratch under a microscope and analyzed with NIH ImageJ software (https://imagej.nih.gov/ij/download.html, accessed on 15 June 2020).

### 2.10. Invasion Assay

Cells (2 × 10^5^) were seeded in 24-transwell chambers with an 8 μm pore membrane coated with Matrigel in the upper chamber of the transwell system containing serum-free RPMI 1640 medium. The lower chamber of the transwell chamber contained a medium with 20% FBS. After incubation at 37 °C for 6 h, the noninvaded HCC cells on the upper side of the membrane were carefully removed with a cotton swab, whereas the invaded cells were stained with crystal violet dye, air dried, and photographed under a microscope. Images were analyzed with ImageJ.

### 2.11. Sphere Formation Assay

Cells (5 × 10^3^ per well) were plated in ultra-low-attachment six-well plates (Corning) containing stem cell medium comprising serum-free RPMI 1640 medium supplemented with 10 ng/mL human basic fibroblast growth factor (bFGF; 1 × B27 supplement, and 20 ng/mL epidermal growth factor (Invitrogen, Grand Island, NY, USA)). The medium was changed every 72 h. After 14 days of incubation, the formed spheres were counted, and photographs were taken.

### 2.12. Animal Studies

All the animal experiments and maintenance conformed to strict compliance with the Animal Use Protocol Taipei Medical University Hospital (protocol LAC-2019-0526). To investigate the anti-proliferative effect of HCQ and sorafenib in combination on HCC cells in vivo, we established a nude mouse model bearing HCC cell xenografts. Five-week-old male athymic nude mice were used for this study. The mice were maintained under pathogen-free conditions and were provided with sterilized food and water. First, 1 × 10^6^ Huh7-SR were injected subcutaneously into the right flank near the hind leg of each nude mouse. When the mice bear palpable tumors (the tumor volume was 100 mm^3^), they were randomly divided into control (100 µL normal saline (NS) by intraperitoneal injection plus 100 µL 1% dimethyl sulfoxide (DMSO)) and 0.5% carboxymethyl cellulose ((CMC)-Na sterile water), HCQ (30 mg/kg/day by intraperitoneal injection plus 100 µL 1% DMSO and 0.5% CMC-Na sterile water)). Sorafenib (10 mg/kg/day by intragastric administration plus 100 µL NS by intraperitoneal injection) and combination (HCQ, 30 mg/kg/day by intraperitoneal injection plus sorafenib 10 mg/kg/day by intragastric administration) groups (n = 6 animals/group) were studied. The treatments were performed for 4 weeks, 5 times per week. The tumor volume was detected every week and was calculated by the following formula: volume = 1/2 (length × width^2^). After 4 weeks, the mice were humanely euthanized, and the tumors were isolated for further analyses.

### 2.13. Statistical Analysis

All assays were performed at least thrice in triplicate. Values are expressed as mean ± standard deviation (SD). Comparisons between groups were estimated using Student’s *t* test for cell line experiments or the Mann–Whitney *U* test for clinical data, Spearman’s rank correlation between variables, and the Kruskal–Wallis test for comparison of three or more groups were conducted. The Kaplan–Meier method was used for survival analysis, and the difference between survival curves was tested with a log-rank test. Univariate and multivariate analyses were performed using a Cox proportional hazards regression model. All statistical analyses were performed using IBM SPSS Statistics for Windows, version 20 (IBM, Armonk, NY, USA). Statistical significance was indicated if *p* < 0.05.

## 3. Results

### 3.1. TLR9 Overexpressed in HCC Sorafenib-Resistant HCC Tissue and Cells

**Figure 1:**
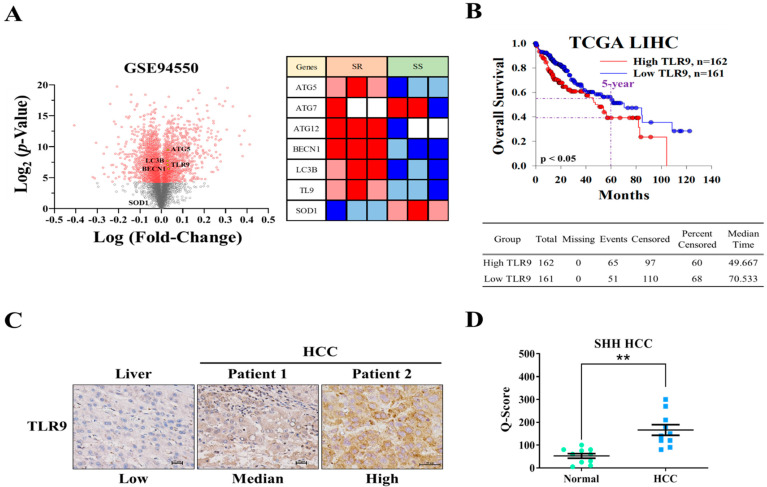
TLR9 was overexpressed in HCC sorafenib-resistant HCC tissue and cells. (**A**) Volcano plot shows differentially expressed genes in sorafenib resistance HCC (Huh7) cells (n = 3) and sensitive cells (n = 3) in GSE94550 GEO datasets. Heatmap illustrates the expression of TLR9 and other important genes associated with HCC sorafenib-resistant cells (right panel). (**B**) Kaplan–Meier curves indicating the correlation of TLR9 expression and the overall 5-year survival of TCGA LIHC patients. (**C**) Immunohistochemistry indicated a higher expression of TLR9 in the HCC tumor tissues. (**D**) Scatter plot reflecting the intensity of TLR9 staining in normal (n = 10) and tumor settings (HCC, n = 10), ** *p* < 0.01. Scale bar: 50 μm.

In an attempt to investigate the role of TLR9 in HCC sorafenib resistance, publically available GEO microarray data (GSE94550) of Huh7 sorafenib resistance (n = 3) and sensitive cells (n = 3) were analyzed showing TLR9, ATG5/7/12, LC3B, and BECN1 were significantly overexpressed, whereas the oxidative stress marker (SOD1 enzyme) expression was reduced in HCC sorafenib-resistant Huh7 cells (Figure 1A). By the analysis of The Cancer Genome Atlas (TCGA) liver hepatocellular carcinoma (LIHC) data, we found that the patients with high TLR9 expression, higher than median level (median time: 49.667), had a poor overall survival when compared with those lower than the median (median time: 70.533) (Figure 1B). Consistently, TLR9 overexpression was further confirmed by immunohistochemical staining for TLR9 in pairs of clinical HCC tissue sections (Figure 1C). Furthermore, the scatter plot showing and confirming that TLR9 was expressed significantly higher in the SHH-HCC tissue cohort (Figure 1D). These results indicate that TLR9 overexpression as distinctly seen is associated with sorafenib resistance in HCC. However, the drug that can target TLR9 in sorafenib-resistant cells still not studied as per our current knowledge. Recent studies have shown the combination of sorafenib and hydroxychloroquine was very effective in treating patients with metastatic liver cancer [45].

### 3.2. Hydroxychloroquine (HCQ) Suppressed the Cell Proliferation and Viability of HCC Cell Lines

**Figure 2:**
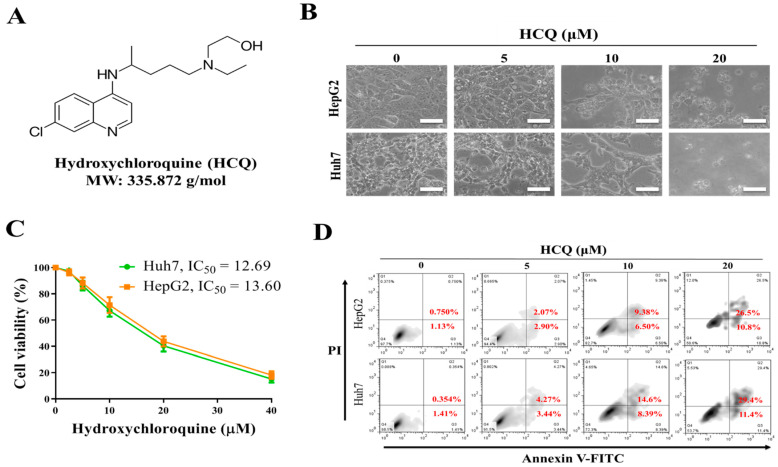
The effect of hydroxychloroquine (HCQ) on cell proliferation and viability of HCC cell lines. (**A**) The chemical structure of HCQ. (**B**) Effect of HCQ on the morphology of the HCC cells (Huh7 and HepG2) shown through the image of the cells taken under the phase-contrast microscopy image at ×200 magnification. Scale bar 100 μm. (**C**) Cytotoxic effect HCQ (IC_50_): Huh7 and HepG2 cells were treated for 96 h with increasing concentration of HCQ (2.5 to 40 μM). SRB assay was performed to analyze cellular viability, DMSO was used as the negative control. (**D**) Apoptosis rate (%) of HCC cells after HCQ treatment was determined using flow cytometry; representative images were taken from HCQ-treated and non-treated control cells stained with annexin V/propidium iodide (magnification ×100).

We investigated the antitumor effects of HCQ on HCC cells. Furthermore, cell viability was investigated using SRB assay to elucidate the effect of HCQ (Figure 2A) treatment on HCC cells (Huh7 and HepG2). Interestingly, after the HCQ treatment for 48 h, all the HCC cells showing inhibition in viability, as shown in Figure 2B, changed in the morphology of cells denoting HCQ treatment, effectively inducing apoptosis on both the cells. The HCQ treatment (48 h) on both the HCC cells showed 50% inhibition in cell viability within the range of 12.69~13.6 μM of HCQ dosage (Figure 2C). The effect of HCQ in inducing the apoptosis of HCC cells was assayed by annexin V/propidium iodide detection kit; as shown in Figure 2D, the treatment of HCQ on both the HCC cells resulted in a significant increase in the ratio of early and late apoptosis cells, while the percentage of viable cells reduced. These results suggest that HCC cells are sensitive to the HCQ treatment.

### 3.3. HCQ Treatment Inhibited Tumor Spheroid Growth and Stem/Self-Renewal Properties of HCC Cell Lines

**Figure 3:**
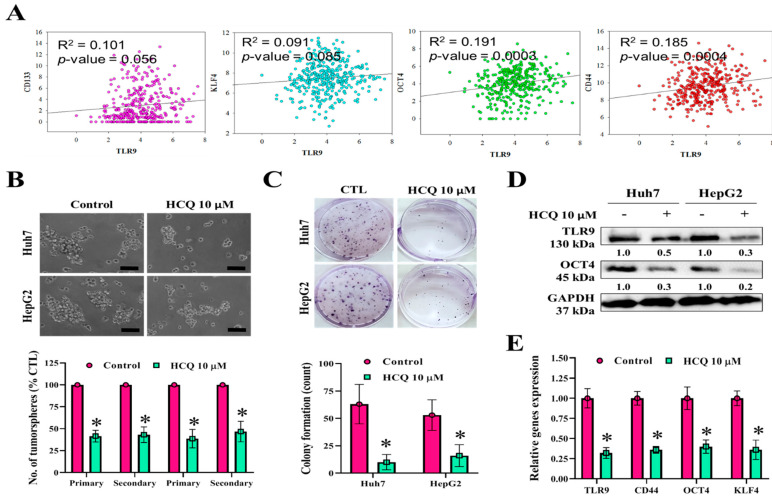
HCQ inhibited HCC cancer stem-like cells’ self-renewal properties. (**A**) Correlation analysis of TLR9 expression with stem cell marker expression. (**B**) HCQ inhibited the tumor sphere-forming capacity (scale bar 200 μm) and (**C**) colony-forming capacity in HCC (Huh7 and HepG2) cells. The bar plot represents the quantification of tumorsphere and colony-forming abilities after HCQ treatment calculated by ImageJ, provided in (B) (below) and (C) (below), respectively. (**D**) Western blot analysis expression of TLR9 and cancer stem/self-renewal-related marker (Oct4) or GAPDH (loading control). Representative protein bands are shown a reduction in expression TLR9 and Oct4 observed after the HCQ treatment (10 μM). (**E**) mRNA expression of TLR9, CD44, Oct44, and KLF4 was measured by qRT-PCR; GAPDH was used to normalize the expression levels. Data are the mean ± SEM of three independent experiments. * *p* < 0.05.

To further examine the effect of HCQ in reducing the stemness and self-renewal properties of HCC cells, we studied the correlation between TLR9 expression and CSCs markers from the TCGA-LIHC database. Scatter plot showed the positive correlation between TLR9 mRNA and CD133 (R^2^ = 0.101), KLF4 (R^2^ = 0.091), OCT4 (R^2^ = 0.191), and CD44 (R^2^ = 0.185) (Figure 3A). Colony-forming and tumorsphere generation are important markers for the identification of stemness [46,47]. As shown in Figure 3B,C, we observed that HCQ treatment resulted in a marked reduction of tumorsphere and colony formatting abilities of HCC cells. Reduction in the expression of tumorsphere and stemness marker (OCT4) and TLR9 expression at the protein level was observed after the HCQ (10 μM) treatment in both the HCC cells (Figure 3D). The qRT-PCR analysis demonstrated at the mRNA level that HCQ treatment significantly reduced the expression of TLR9, CD44, OCT4, and KLF4 in HCC cells (Figure 3E). These results indicate that the stem cell-like phenotype of HCC cells was modulated by the inhibition of expression TLR9 by HCQ treatment.

### 3.4. HCQ Inhibited Oncogenic Potential by Reducing Migration and Invasion of HCC Cells

**Figure 4:**
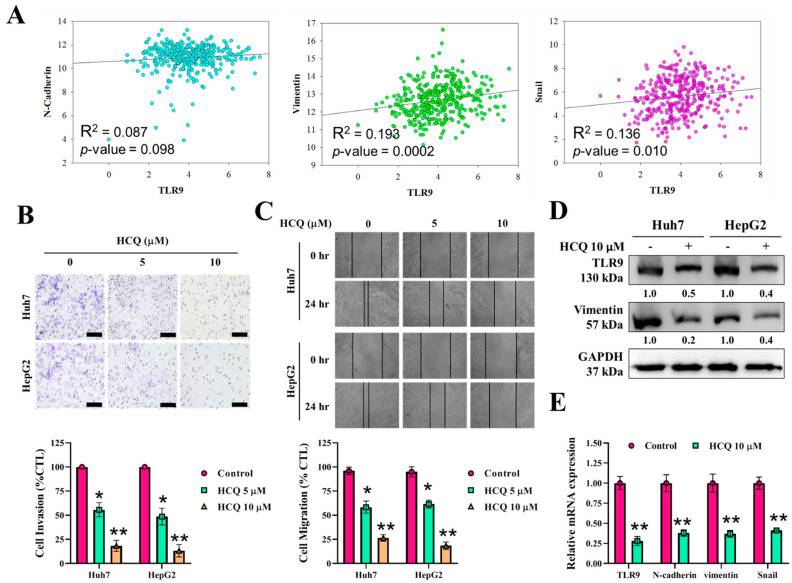
HCQ inhibited HCC cancer cells’ oncogenic and invasive properties. (**A**) Correlation analysis of TLR9 expression with EMT associate marker expression. (**B**,**C**) HCQ significantly inhibited HCC (Huh7 and HepG2) cell invasive and migratory abilities. The representative bar plot represents the quantification of invasion and migratory abilities after HCQ treatment calculated by ImageJ, provided in (**B**) (below) and (**C**) (below), respectively. (**D**) Western blot analysis of expression of TLR9, and EMT marker (vimentin); GAPDH was used as an internal control. Representative protein bands are shown a reduction in expression of TLR9 and vimentin observed after the HCQ treatment (10 μM). (**E**) The mRNA expression level of TLR9 and EMT markers (N-cadherin, vimentin, and Snail2) were determined using real-time qRT-PCR; GAPDH was used to normalize the expression levels. Data are the mean ± SEM of three independent experiments. * *p* < 0.05, ** *p* < 0.01.

We aimed to determine whether HCQ inhibits the oncogenic potential of HCC cells. The correlation analysis of TLR9 mRNA expression, as shown in Figure 4A, positively correlated with the expression EMT biomarkers such as N-cadherin (R^2^ = 0.087), vimentin (R^2^ = 0.193), and snail (R^2^ = 0.136). Under the HCQ (5~10 μM) treatment for 48 h, the invasive (Figure 4B) and migratory (Figure 4C) abilities of the HCC cells were greatly inhibited, indicating that HCQ effectively reduced the invasiveness and mobility of the HCC cells, as compared to their untreated control counterparts. EMT has been associated with invasion/cell mobility and metastasis of HCC [48]. The expression EMT markers and TLR9 after the HCQ treatment were determined by qRT-PCR and Western blot analysis. As depicted in Figure 4D,E, TLR9 and EMT markers such as vimentin, N-cadherin, and snail were significantly decreased in the HCQ treatment HCC cells group. These results suggest that HCQ is effective in preventing HCC cell migration and invasion through modulating the expression of TLR9.

### 3.5. HCQ Modulated the Expression of Oxidative Stress Marker (SOD1) in HCC Cells

**Figure 5:**
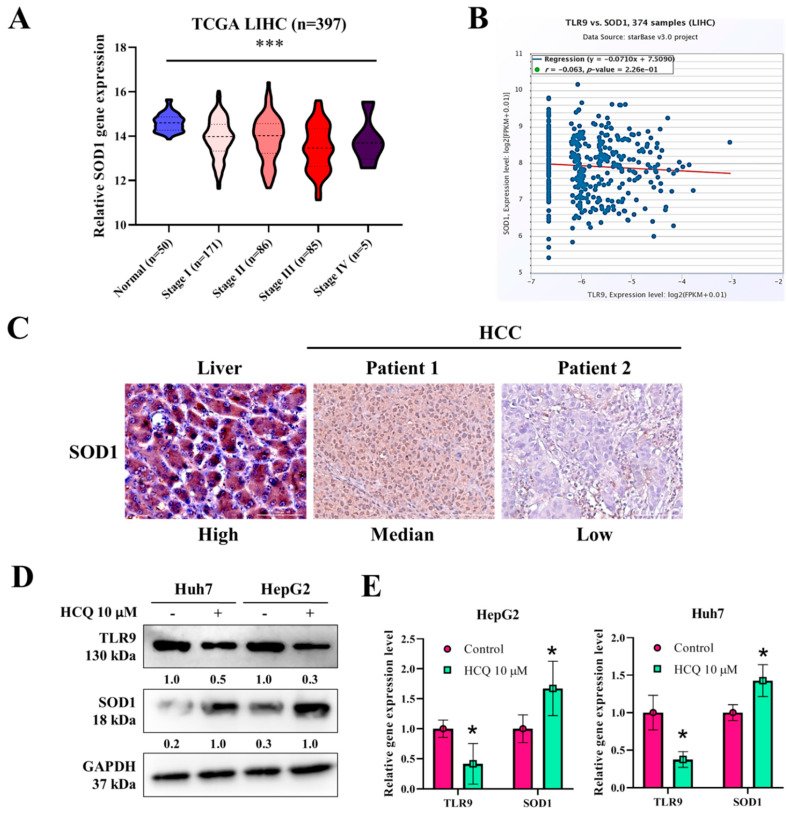
HCQ treatment modulated the expression of antioxidant enzyme expression in HCC cells. (**A**) SOD1 expression in TCGA-LIHC patients at higher grade compared to normal samples, analyzed using online UALCAN tool; http://ualcan.path.uab.edu/cgi-bin/ualcan-res.pl (accessed on 15 June 2020). (**B**) Correlation analysis of TLR9 expression with SOD1 expression in TCGA-LIHC samples analyzed using online starBase v3.project tool; http://starbase.sysu.edu.cn/ (accessed on 15 June 2020). (**C**) Immunohistochemistry indicated a reduced expression of TLR9 in the HCC tumor tissues. (**D**) Western blot and qRT-PCR analysis expression of TLR9 and SOD1, or GAPDH (loading control). Representative protein bands are shown a reduction in expression of TLR9 and SOD1 observed after the HCQ treatment (10 μM). (**E**) mRNA expression of TLR9 and SOD1 was measured by qRT-PCR; GAPDH was used to normalize the expression levels. Data are the mean ± SEM of three independent experiments. * *p* < 0.05, *** *p* < 0.001.

Furthermore, we analyzed the expression of SOD1 in TCGA-LIHC samples and cell lines. SOD1 expression was significantly reduced in LIHC higher-grade tumor samples as compared to the normal tissues (*p* < 2.319400 × 10^−3^) (Figure 5A). The correlation analysis of TLR9 mRNA expression, as shown in Figure 5B, negatively correlated with the expression of SOD1 (R^2^ = −0.063). Consistently, the observation of SOD1 expression being reduced was further confirmed by immunohistochemically staining for SOD1 in a pair of clinical HCC tissue sections (Figure 5C). Interestingly, after the HCQ treatment on both the HCC cells, the expression of SOD1 at the protein and mRNA levels was significantly induced as determined by Western blot and qRT-PCR analysis (Figure 5D,E). These results suggest that HCQ is effective in inducing the expression of SOD1 in HCC cells through modulating the TLR9 expression.

### 3.6. HCQ Displayed a Synergistic Effect with the Sorafenib on HCC Sorafenib-Resistant Cells

**Figure 6:**
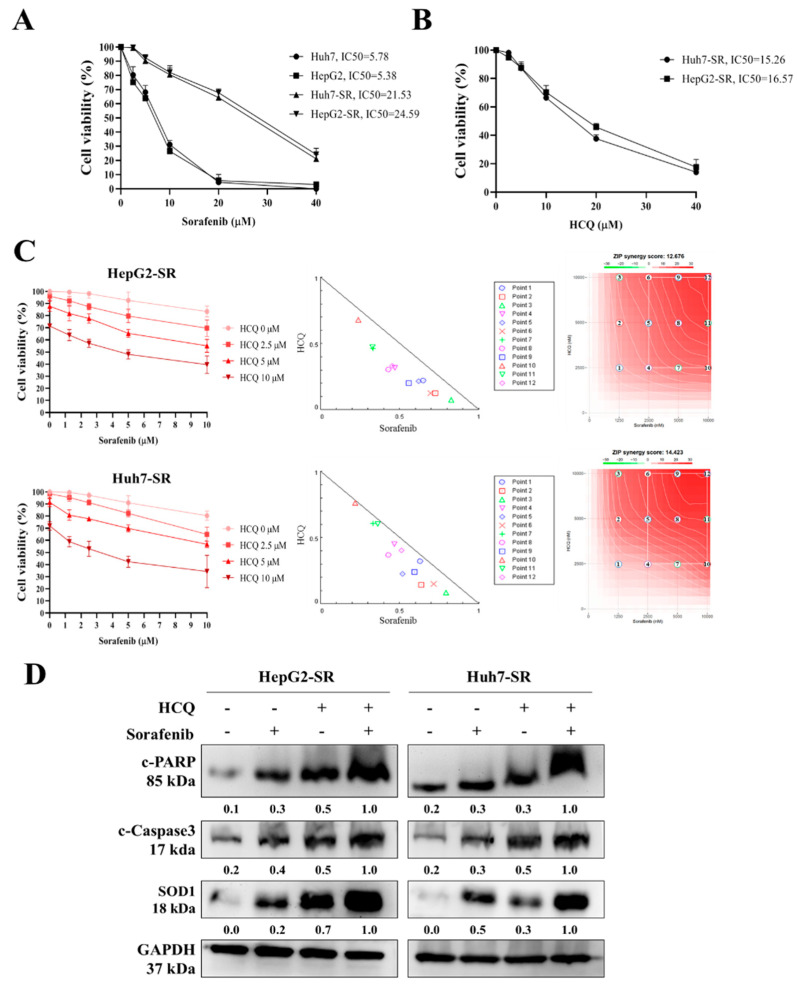
HCQ treatment sensitized sorafenib-resistant HCC cells towards sorafenib treatment. (**A**) Huh7, Huh7-SR, HepG2, and HepG2-SR cells were treated with sorafenib for 48 h. Cell viability (%) was measured and normalized with the control untreated cells. (**B**) HCQ dose-dependently reduced the viability of sorafenib-resistant HCC cell lines (right panel). (**C**) Isobologram analysis represents the combined effect of HCQ and sorafenib on sorafenib-resistant HCC cells and growth inhibitory effect of HCQ and sorafenib on HCC cells after the 48-h exposure. CI < 1 indicates synergism. (**D**) Cells were treated with HCQ, sorafenib individually or in combination, Western blot to detect the cleavage of caspase-3 and PARP, and SOD1 expression. GAPDH was used as the control for equal loading.

Sorafenib has been seen to significantly improve the survival of HCC patients, but frequently these patients develop resistance against it [49]. In an attempt to investigate the mechanism of sorafenib resistance in HCC, we introduced an in vitro model by culturing sorafenib-resistant cell lines with long-term exposure to sorafenib in the culture medium as per the methods described by Van Malenstein et al. (2013) with slight modification [50]. Resistance is considered to be achieved when cells can withstand a higher concentration of sorafenib than that of the parental cell line. Two HCC-resistant cell lines (Huh7-SR and HepG2-SR) were established. Resistant cell lines showed higher cell viability (high IC_50_) in the presence of sorafenib than that of parental cell lines (Figure 6A). Furthermore, we tested the HCQ effect on the viability of sorafenib-resistant cells, observing a slight increase in the IC_50_ values in sorafenib-resistant cells compared to parental cells (Figure 6B). Thus, we tested whether the combination of HCQ and sorafenib can suppress HCC cell proliferation towards the sorafenib in sorafenib-resistant cell lines (Huh7-SR and HepG2-SR). Interestingly, isobologram analysis showed that the combined effect of HCQ with sorafenib treatment synergistically inhibited and re-sensitized HCC-resistant cell proliferation (CI values < 1) to sorafenib, as shown in Figure 6C, and induced the expression of apoptotic markers such as c-PARP, c-caspase-3, and SOD1 in sorafenib-resistant cells (Figure 6D). These results denote the combination of HCQ–sorafenib can overcome the sorafenib resistance of HCC cells.

### 3.7. HCQ Treatment Modulated the Autophagy and Apoptosis of HCC Sorafenib-Resistant Cells In Vitro

**Figure 7:**
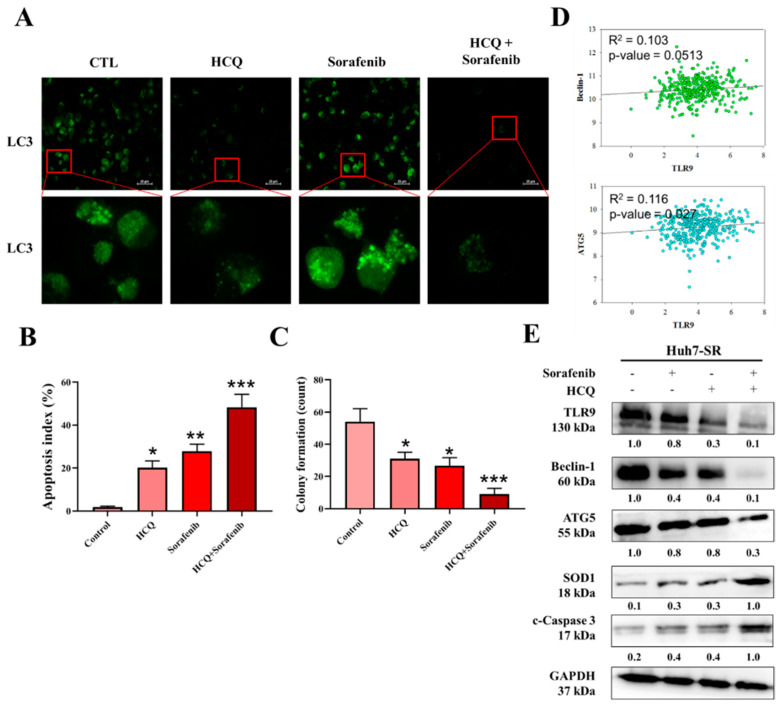
HCQ and sorafenib had a dual treatment effect and sensitized HCC sorafenib-resistant cells in vitro. (**A**) Autophagy body formation was assayed by fluorescence microscopy after staining. (**B**) FACS profile of annexin V/7-AAD staining of Huh7-SR cells undergoing apoptosis induced by HCQ (5 µM) and sorafenib alone (5 µM) or in combination (both the agents at 5 µM). (**C**) Reduction in self-renewal ability, a significant reduction in the colony-forming abilities observed in combination treatment. (**D**) Correlation analysis of TLR9 expression with autophagy-related marker expression (R^2^ = 0.103 in Beclin-1 and R^2^ = 0.116 in ATG5). (**E**) HCQ and sorafenib cotreatment against TLR9, ATG5, Beclin-1, SOD1, and c-caspase-3 expression levels were measured by Western blotting: * *p* < 0.05, ** *p* < 0.01, *** *p* < 0.001.

Autophagy is well known to modulate a cancer cell’s growth depending upon the cell status. Previously, sorafenib was reported to induce autophagy and apoptosis that could be a key mechanism for the development of sorafenib resistance in HCC. First, we evaluated the potential autophagic effect of HCQ and sorafenib in sorafenib-resistant Huh7-SR HCC cells. The expression of LC3 is a key indicator of showing the formation of autophagosome. As depicted in Figure 7A, the treatment of HCQ (5 µM), sorafenib individually (5 µM) or in combination (5 µM concentration of both the agents), and HCQ treatment either alone or in combination with sorafenib results in the significant reduction of formation of autophagosome bodies (reduced LC-3 expression). Furthermore, apoptosis and colony-forming abilities of Huh7-SR cells were analyzed by the FACS analysis of annexin V/7-AAD staining and SRB assay. As demonstrated in Figure 7B,C, the HCQ–sorafenib combination-treated cells showed significantly induced apoptosis and reduced colony-forming abilities, respectively. Interestingly, the expression of essential markers of autophagosome (ATG5 and Becline-1) positively correlated with the expression of TLR9 mRNA expression (Figure 7D). At the same time HCQ only or in combination with sorafenib significantly reduced the protein expression of TLR9, ATG5, and Beclin-1, and induced expression of the antioxidative enzyme (SOD1) and apoptotic (c-caspase-3) marker (Figure 7E) was observed. Notably, HCQ–sorafenib-induced anti-HCC effect correlated with autophagy cell death.

### 3.8. MiR-30a-5p Regulated the Expression of Autophagy Cell Markers in Sorafenib-Resistant HCC Cells

**Figure 8:**
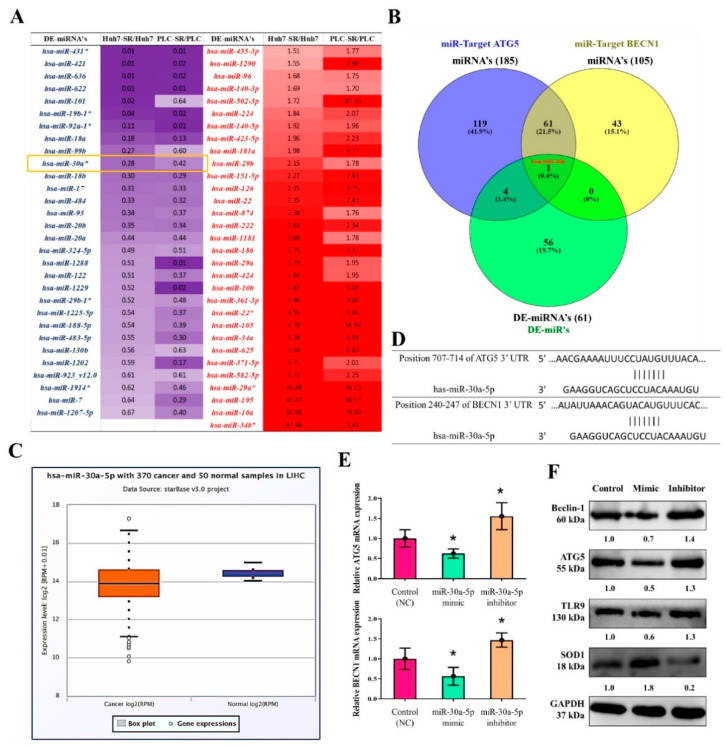
miR-30a-5p-regulated the expression of autophagy cell markers in sorafenib-resistant HCC cells. (**A**) Heatmap for 61 differentially expressed miRNA sorafenib-resistant cells. (**B**) miRNA target analysis of the ENCORI database revealed 185 ATG5 and 105 Beclin-1 miRNA targets. (**C**) hsa-miR-30a-5p expression in TCGA-LIHC samples analyzed using starBase v3 project tool; http://starbase.sysu.edu.cn/ (accessed on 15 June 2020). (**D**) TargetScan database indicated that hsa-miR-30a-5p predictively bound to ATG5 and Beclin-1. (**E**,**F**) Effect of hsa-miR-30a-5p overexpression (mimic) and inhibition (inhibitor) on the mRNA and protein expressions of ATG5, Beclin-1, TLR9, and SOD1 in sorafenib-resistant HCC (Huh7-SR) cells. * *p* < 0.05.

Noncoding RNAs, especially miRs, play a key role in regulating the outcome of and resistance to therapy in HCC [51,52]. We analyzed miRNA expression data from sorafenib-resistant (Huh7-SR and PLC-SR) versus its parental variant (Huh7 and PLC) from the study by Xu et al. [7]. The heatmap indicated that 61 differentially expressed miRNAs were significantly upregulated or downregulated in the sorafenib-resistant cells relative to parental cells (Figure 8A). A miRNA target analysis of the ENCORI database [53,54] revealed 185 ATG5 and 105 Beclin-1 miRNA targets (Figure 8B). Furthermore, an intersection analysis (Venn diagram, Figure 8B) revealed that hsa-miR-30a-5p was a key miRNA targeting both ATG5 and Beclin-1 and was significantly downregulated in the resistant cells compared with parental cells. The expression of hsa-miR-30a-5p was significantly reduced in LIHC tumor samples in comparison with normal tissues (Figure 8C). We then searched for hsa-miR-30a-5p targets and identified them through the TargetScan database [55], which indicated that hsa-miR-30a-5p predictively binds to ATG5 and Beclin-1 (Figure 8D). The effect of hsa-miR-30a-5p on sorafenib-resistant HCC (Huh7-SR) cells was evaluated using gain-and-loss-of-function experiments (Figure 8E,F). hsa-miR-30a-5p overexpression significantly inhibited ATG5 and Beclin-1 expression after transfection with hsa-miR-30a-5p (mimic) in comparisons with the control group, whereas hsa-miR-30a-5p inhibition cells significantly elevated ATG5 and Beclin-1 mRNA and protein expression. Notably, TLR9 expression and SOD1 level were also modulated due to hsa-miR-30a-5p inhibition/overexpression.

### 3.9. In Vivo Effect of HCQ Treatment Sensitized Sorafenib Efficacy on Sorafenib-Resistant HCC Cells

**Figure 9:**
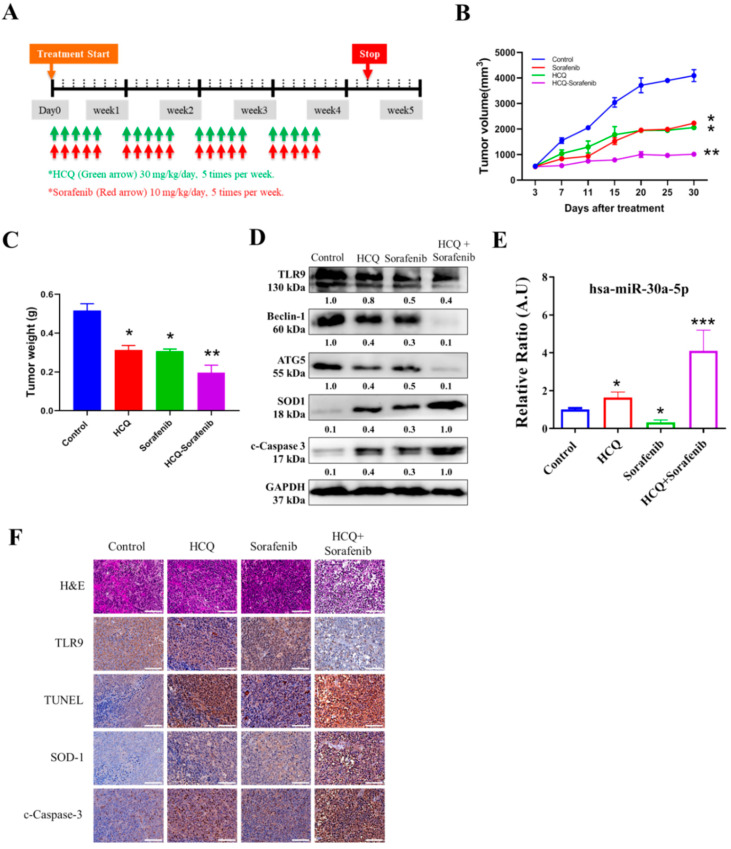
In vivo effect of HCQ treatment sensitizes sorafenib efficacy on sorafenib-resistant HCC cells. Effect of combination therapy on the in vivo growth of HCC (Huh7-SR) cancer cells. (**A**) HCQ/sorafenib and combination treatment administration. (**B**,**C**) Representative images and bar plot of Huh7-SR xenograft tumor volume and weight between control and combination drug treatment groups. (**D**) Western blot analysis of expression of TLR9, autophagosome markers (ATG5 and Beclin-1), apoptotic marker (c-caspase-3), and antioxidative enzyme SOD1 expression, or GAPDH (loading control). (**E**) qRT-PCR analysis of expression of hsa-miR-30a-5p in blood serum collected from animals. (**F**) Image representative of hematoxylin and eosin (H&E) staining; TdT-mediated dUTP nick-end labelling (TUNEL) assay; and the results of c-caspase-3, SOD-1, and TLR9 by immunohistochemistry (IHC). * *p* < 0.05, ** *p* < 0.01, *** *p* < 0.001.

After establishing in vitro HCQ’s anti-HCC role, for our in vivo study, we evaluated the HCQ alone or in combination with sorafenib effects using a xenograft mouse Huh7-SR tumor model. The mice were treated with the indicated dose of HCQ, sorafenib, or combination, as scheduled (Figure 9A). The tumor volume over time clearly showed that HCQ treatment together with sorafenib combination resulted in significantly delayed tumorigenesis, while the vehicle and HCQ and sorafenib alone groups displayed no significant modulation of tumor volume and weight (Figure 9B,C). Comparatively, Western blots from tumor samples collected in all groups demonstrated a significant reduction in the expression of TLR9, autophagosome markers (ATG5 and Beclin-1), and induction in the expression of apoptotic marker (c-caspase-3) and oxidative enzyme (SOD1) expression observed (Figure 9D), while the qRT-PCR analysis of plasma levels of hsa-miR-30a-5p expression showed the highest level in HCQ + sorafenib-treated pooled blood samples, followed by HCQ, control, and sorefenib (Figure 9E). As expected, the HCQ–sorafenib combination treatment results, as described from the hemotoxylin and eosin (H&E) staining, TdT-mediated dUTP nick-end labelling (TUNEL) assay and analyzed by immunohistochemistry (IHC). IHC analysis of tissue sections exhibited significant changes in morphology, with the sign of necrosis with the inflammatory cell’s invasion and fibrosis (Figure 9F, upper panel). A combination of HCQ–sorafenib showed a minimal increase in the total number of TUNEL-positive cells (Figure 9F, middle panel). Additionally, combination treatment also indicated an effective induction of apoptotic marker (c-caspase-3) protein levels in comparison with those treated with HCQ and sorafenib alone (Figure 9F, bottom panel). Our findings suggest that HCQ–sorafenib potently enhances and re-sensitizes sorafenib towards sorafenib-resistant HCC.

## 4. Discussion

HCC is a major cause of cancer-related death worldwide [56], with a 5-year survival rate of <18%. Since the clinical approval of sorafenib, it has been used frequently as a part of molecular targeted therapy for treating advanced stages of HCC [6]. However, some patients with HCC who initially responded to sorafenib [7] experience therapy failure later due to the rapid acquisition of chemoresistance, which remains a key obstacle to successful cancer therapy. The key molecular mechanism underlying sorafenib resistance in HCC remains poorly characterized.

One of the reasons for sorafenib resistance is the involvement of ROS, which are indispensable in several normal physiological processes of cell proliferation, viability, apoptosis, and senescence and cell cycle arrest [11]. However, increased oxidative stress creates a potentially toxic environment [12], causing free radical damage to the DNA, which is neutralized by SOD and CAT [12]. Because SOD1 is the major type of SOD located in the mitochondrial intermembrane space [12,15], its impairment might contribute to cancer tumorigenesis [16]. In this study, we examined the cellular alterations and molecular mechanisms underlying acquired sorafenib resistance in HCC.

The key events resulting in HCC are chronic inflammation, hepatic fibrosis, and immune activation by gut bacteria translocation. Moreover, both pathogen-related and damage-related molecular patterns, such as the roles of PAMPS and DAMPS, are increased in chronic liver disease, resulting in liver hyperinflammation [57]. TLRs, especially TLR7 and TLR9, are upregulated in HCC [21]. HCC cells exhibit increased proliferation under the stimulation of TLR9, whereas the inhibition of TLR9 using HCQ results in the decrease of HCC proliferation and tumor growth both in vitro and in vivo [21].

A few miRNAs, such as miR-222, have been associated with the regulation of sorafenib resistance or sensitivity in cancer chemotherapy [26,27] along with autophagy [28]. Sorafenib induces both autophagy and apoptosis, which could be a key mechanism for the development of sorafenib resistance in HCC [28]. TLR9 overexpression was observed in sorafenib-resistant HCC cells and clinical samples; this was related to the poor overall survival of patients with sorafenib-resistant HCC (Figure 1). This suggested that TLR9 can be a novel target to overcome sorafenib resistance. TLRs expression essentially has been reported to create a connection between host immune response against these infections. In cancers, after the tumor cells die, they release a number of proteic or nuclear DAMPs that result in the induction of TLRs expression on both the immune/stromal and epithelial cancer cells, indicating TLR works as a double-edged sword, either promoting or inhibiting tumor progression [58]. More importantly, the role of TLR (toll-like receptor), especially TLR7 and TLR9 expression, was observed to be upregulated in HCC [25]. HCC cells show increased proliferation under the stimulation of TLR9, whereas inhibition of TLR9 using HCQ results in the reduction of HCC proliferation and tumor growth both in vitro and in vivo [25]. Considering the anti-cancer properties of HCQ, clinical studies have provided evidence for the repurposing of HCQ for use in cancer treatment. Approximately 30 clinical studies have evaluated the anticancer properties of HCQ in many cancer types with a combination of standard treatments [34]. Its roles in inhibiting autophagosome–lysosome fusion (LC3 cell marker, ATG5, and Beclin-1 expression) and reducing chemoresistance has gained considerable attention [37]. CSCs and their self-renewal properties have been associated with the development of chemoresistance in HCC; CSCs “hijack” angiogenesis in vasculogenic imitation [41]. Colony-forming and tumorsphere generation are key markers of stemness [46,47]. EMT has been associated with invasion and cell mobility and metastasis in HCC [48]. Our study’s findings have consistently demonstrated that HCQ treatment effectively targeted TLR9 expression, resulting in reduced HCC cell proliferation and induced apoptosis (Figure 2). HCQ treatment also significantly targeted the stem cells and EMT, reduced the formation of the tumorsphere, and inhibited colony-forming abilities (self-renewal) (Figure 3). Furthermore, HCQ treatment reduced the oncogenic potential of HCC cells by inhibiting the EMT process—that is, by diminishing the migratory and invasive properties of HCC cells (Figure 4). Notably, HCQ effectively enhanced and protected the cells from oxidative DNA damage stress by enhancing the expression of the antioxidant SOD1 gene in HCC cells (Figure 5) to protect the cells from oxidative DNA damage.

As mentioned, because patients with HCC often develop sorafenib resistance, combined therapy targeting sorafenib resistance mechanisms is required to overcome it. Therefore, we developed an in vitro model of sorafenib-resistant cell lines [50]. Combination therapy has become widely popular in cancer treatment because it targets multiple pathways with different mechanisms, thereby decreasing the development of chemoresistance [59]. The combinations of two therapeutic agents can produce a synergistic (CI < 1), antagonistic (CI > 1), or additive (CI = 1) effect. In our study, the HCQ–sorafenib combination treatment resulted in a decrease in the proliferation of sorafenib-resistant HCC cells and an increase in the levels of antioxidant and apoptotic markers (Figure 6). Furthermore, the combination-treated Huh7-SR cells exhibited decreased autophagosome formation and colony-forming ability and increased apoptosis (Figure 7). The combination-treated cells also exhibited a decrease in the protein expression of autophagosome expression markers, such as ATG5 [28], Beclin-1 [28], and TLR9.

The bioinformatics analysis revealed the role of epigenetic factors in controlling the severity of sorafenib resistance in HCC cells. We observed that hsa-miR-30a-5p was downregulated in TCGA-LIHC tumor, sorafenib-resistant HCC Huh7, and PALC cells. Predictive binding, gain-of-function (overexpression), and loss-of-function (inhibition) analyses verified the importance of hsa-miR-30a-5p in modulating ATG5 and Beclin-1 expression in vitro (Figure 8). The inhibition of hsa-miR-30a may retard tumor progression by decreasing Beclin-1 and ATG5 expression and inhibiting autophagy, thereby sensitizing the tumor cells to the drug therapy [59,60]. *In vivo* study also supported these observations that HCQ alone or with sorafenib results in sensitization to sorafenib treatment and that the HCQ–sorafenib combination treatment significantly induced has-miR-30a-5p expression. Moreover, cleaved caspase-3 staining and TUNEL assay revealed that the combination treatment upregulated *SOD1* expression and apoptosis in xenograft tumors (Figure 9).

## 5. Conclusions

**Figure 10:**
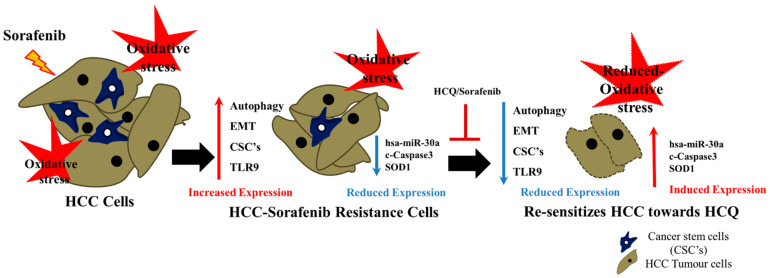
HCQ–sorafenib combined therapy modulated TLR9 expression, downregulated the expression of cancer stemness genes, and enhanced the antitumor efficacy of sorafenib; it did so by inducing the expression of antioxidant SOD1 enzymes and by reducing oxidative DNA damage and levels of apoptosis-associated genes in sorafenib-resistant HCC cells via the has-miR-30a-5p axis, thus maximizing the therapeutic potential for treating patients with sorafenib-resistant HCC.

Our results revealed that sorafenib resistance in HCC is a complex process (Figure 10), with many key factors affecting the efficacy of therapy. Both *in vitro* and *in vivo* findings indicated that TLR9 overexpression was correlated with CSCs, EMT, antioxidant SOD1 enzyme, and autophagosome expression markers. However, the HCQ–sorafenib combination reduced TLR9 expression, downregulated the expression of cancer stem genes, and enhanced the antitumor efficacy of sorafenib by inducing SOD1 expression and reducing oxidative DNA damage and apoptosis-associated genes in sorafenib-resistant HCC cells via the has-miR-30a-5p axis. Together, these mechanisms maximize the therapeutic potential for treating patients with sorafenib-resistant HCC.

## Data Availability

The datasets used and analyzed in the current study are publicly accessible, as indicated in the manuscript.

## Supplementary Materials

The following are available online at https://www.mdpi.com/article/10.3390/cancers13133227/s1, Table S1. The membranes were incubated in primary antibodies. Western blot raw data: Figure S1. Full-size blots of Figure 3D. Figure S2. Full-size blots of Figure 4D. Figure S3. Full-size blots of Figure 5D. Figure S4. Full-size blots of Figure 6D. Figure S5. Full-size blots of Figure 7E. Figure S6. Full-size blots of Figure 8F. Figure S7. Full-size blots of Figure 9D.
