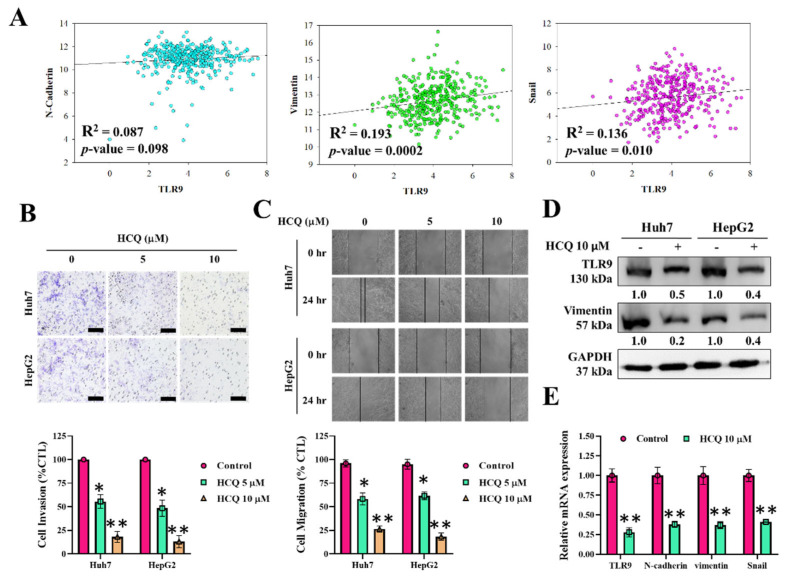# Correction: Chen et al. Hydroxychloroquine (HCQ) Modulates Autophagy and Oxidative DNA Damage Stress in Hepatocellular Carcinoma to Overcome Sorafenib Resistance via TLR9/SOD1/hsa-miR-30a-5p/Beclin-1 Axis. *Cancers* 2021, *13*, 3227

**DOI:** 10.3390/cancers15041028

**Published:** 2023-02-06

**Authors:** Ming-Yao Chen, Vijesh Kumar Yadav, Yi Cheng Chu, Jiann Ruey Ong, Ting-Yi Huang, Kwai-Fong Lee, Kuen-Haur Lee, Chi-Tai Yeh, Wei-Hwa Lee

**Affiliations:** 1 Division of Gastroenterology and Hepatology, Department of Internal Medicine, School of Medicine, College of Medicine, Taipei Medical University, Taipei 110, Taiwan; 2 Division of Gastroenterology and Hepatology, Department of Internal Medicine, Shuang Ho Hospital, New Taipei City 23561, Taiwan; 3 Department of Medicine, St. George’s University School of Medicine, St. George SW17 0RE, Grenada; 4 Department of Emergency Medicine, Taipei Medical University-Shuang Ho Hospital, New Taipei City 23516, Taiwan; 5 Department of Emergency Medicine, School of Medicine, Taipei Medical University, Taipei 110, Taiwan; 6 Biobank Management Center, Taipei Medical University-Shuang Ho Hospital, New Taipei City 23561, Taiwan; 7 Graduate Institute of Cancer Biology and Drug Discovery, College of Medical Science and Technology, Taipei Medical University, Taipei 110, Taiwan; 8 Cancer Center, Wan Fang Hospital, Taipei Medical University, Taipei 110, Taiwan; 9 Department of Medical Research & Education, Taipei Medical University Shuang Ho Hospital, New Taipei City 23561, Taiwan; 10 Department of Medical Laboratory Science and Biotechnology, Yuanpei University of Medical Technology, Hsinchu 300, Taiwan; 11 Department of Pathology, Taipei Medical University Shuang Ho Hospital, New Taipei City 23561, Taiwan

**Error in Figure**

The authors wish to make the following correction to this paper [1]. Due to the mishandling, authors inserted the wrong image of GAPDH in Figure 4D; therefore, the authors replaced the GAPDH image in Figure 4D. The corrected image for Figure 4 is as below:

The authors would like to apologize for any inconvenience caused to the readers by these changes. The original publication has also been updated.

## Figures and Tables

**Figure:**