# Supplementary material for: Hydroxychloroquine (HCQ) Modulates Autophagy and Oxidative DNA Damage Stress in Hepatocellular Carcinoma to Overcome Sorafenib Resistance via TLR9/SOD1/hsa-miR-30a-5p/Beclin-1 Axis
Source: Cancers (Basel). 2021 Jun 28;13(13):3227. doi: 10.3390/cancers13133227 (PMC8267639; doi:10.3390/cancers13133227)
Supplement: Supplementary file 1 [file cancers-13-03227-s001.zip › cancers-1207641-supplementary.pdf]

Supplementary Data

Hydroxychloroquine (HCQ) modulates autophagy and oxidative DNA damage stress in hepatocellular carcinoma to overcome sorafenib-resistant via TLR9/SOD1/hsa-miR-30a-5p/Beclin-1 axis

Ming-Yao Chen [1, 2,] Vijesh Kumar Yadav [1,2,] Yi Cheng Chu [3,] Jiann Ruey Ong [4,5,] Ting-Yi Huang [6,] Kwai-Fong Lee [6,] Kuen-Haur Lee [7,8*,] Chi-Tai Yeh [9,10*] and Wei-Hwa Lee [11]

1 Division of Gastroenterology and Hepatology, Department of Internal Medicine, School of Medicine, College of Medicine, Taipei Medical University, Taipei 110, Taiwan; 08350@s.tmu.edu.tw (M.-Y.C.); 20604@s.tmu.edu.tw (V.K.Y.)

2 Division of Gastroenterology and Hepatology, Department of Internal Medicine, Shuang Ho Hospital, New Taipei City 23561, Taiwan

3 Department of Medicine, St. George's University School of Medicine, St. George SW17 0RE, Grenada; ychu@sgu.edu

4 Department of Emergency Medicine, Taipei Medical University - Shuang Ho Hospital, New Taipei City, Taiwan; malsia95@gmail.com (J.R.O.); 15729@s.tmu.edu.tw (T.-Y.H.)

5 Department of Emergency Medicine, School of Medicine, Taipei Medical University, Taipei, Taiwan.

6 Biobank management center, Taipei Medical University-Shuang Ho Hospital, New Taipei City 23561, Taiwan; 19118@s.tmu.edu.tw

7 Graduate Institute of Cancer Biology and Drug Discovery, College of Medical Science and Technology, Taipei Medical University, Taipei 110, Taiwan;

8 Cancer Center, Wan Fang Hospital, Taipei Medical University, Taipei 110, Taiwan

9 Department of Medical Research & Education, Taipei Medical University Shuang Ho Hospital, New Taipei City 23561, Taiwan.

10 Department of Medical Laboratory Science and Biotechnology, Yuanpei University of Medical Technology, Hsinchu 300, Taiwan

11 Department of Pathology, Taipei Medical University Shuang Ho Hospital, New Taipei City 23561, Taiwan; whlpath97616@s.tmu.edu.tw

* Correspondence:

Dr. Kuen-Haur Lee, Ph.D.

Graduate Institute of Cancer Biology and Drug Discovery, College of Medical Science and Technology, Taipei Medical University, Taipei, Taiwan. Email: khlee@tmu.edu.tw Tel.: +886-2-2490088 ext. 8881

Dr. Chi-Tai Yeh, Ph.D

Department of Medical Research & Education, Taipei Medical University - Shuang Ho Hospital, New Taipei City, 235, Taiwan. No. 291, Zhongzheng Road, Zhonghe District, New Taipei City, Taiwan 235. Email: ctyeh@s.tmu.edu.tw Tel.: +886-2-2490088 ext. 8885

Supplementary Table S1. The membranes were incubated in primary antibodies

No.	Target	Dilution	Source	
1	TLR9	1:200	sc-6248	Santa Cruz
2	OCT4	1:200	sc-23896	Santa Cruz
3	GAPDH	1:200	sc-7961	Santa Cruz
4	Vimentin	1:500	#2978	Cell Signaling
5	c-PARP	1:500	#4129	Cell Signaling
6	Beclin1	1:500	#4656	Cell Signaling
7	ATG5	1:500	#80312	Cell Signaling
8	PARP	1:1000	#9532	Cell Signaling
9	BRCA1	1:1000	#9010	Cell Signaling
14	c-Caspase3	1:1000	ab13847	Abcam
15	Bak	1:2000	ab32371	Abcam
16	Bax	1:2000	ab32503	Abcam
17	Bcl2	1:2000	ab32124	Abcam
18	GAPDH	1:10000	10494-1-AP	proteintech

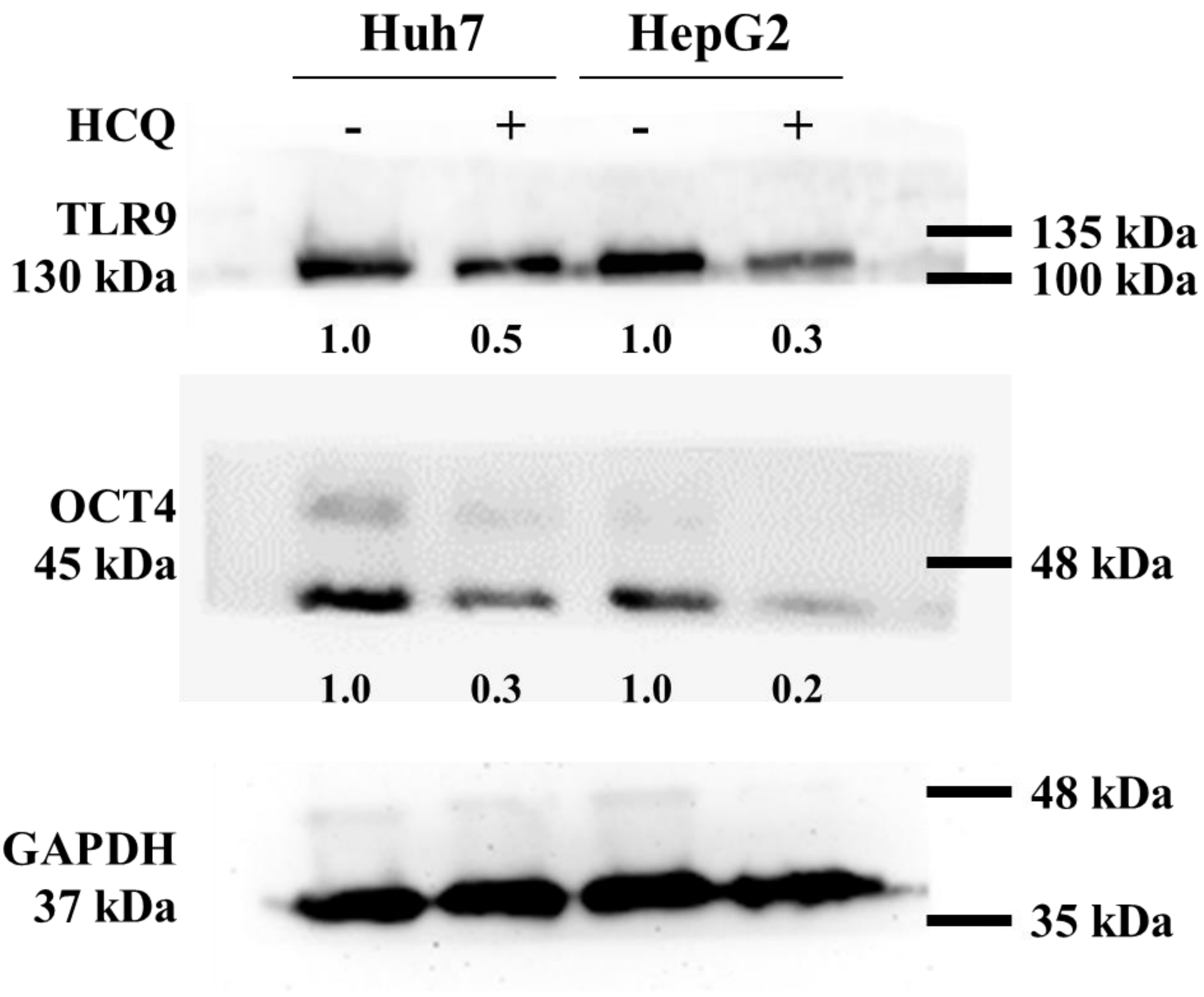

Supplementary Figure S1. Full-size blots of Figure 3D

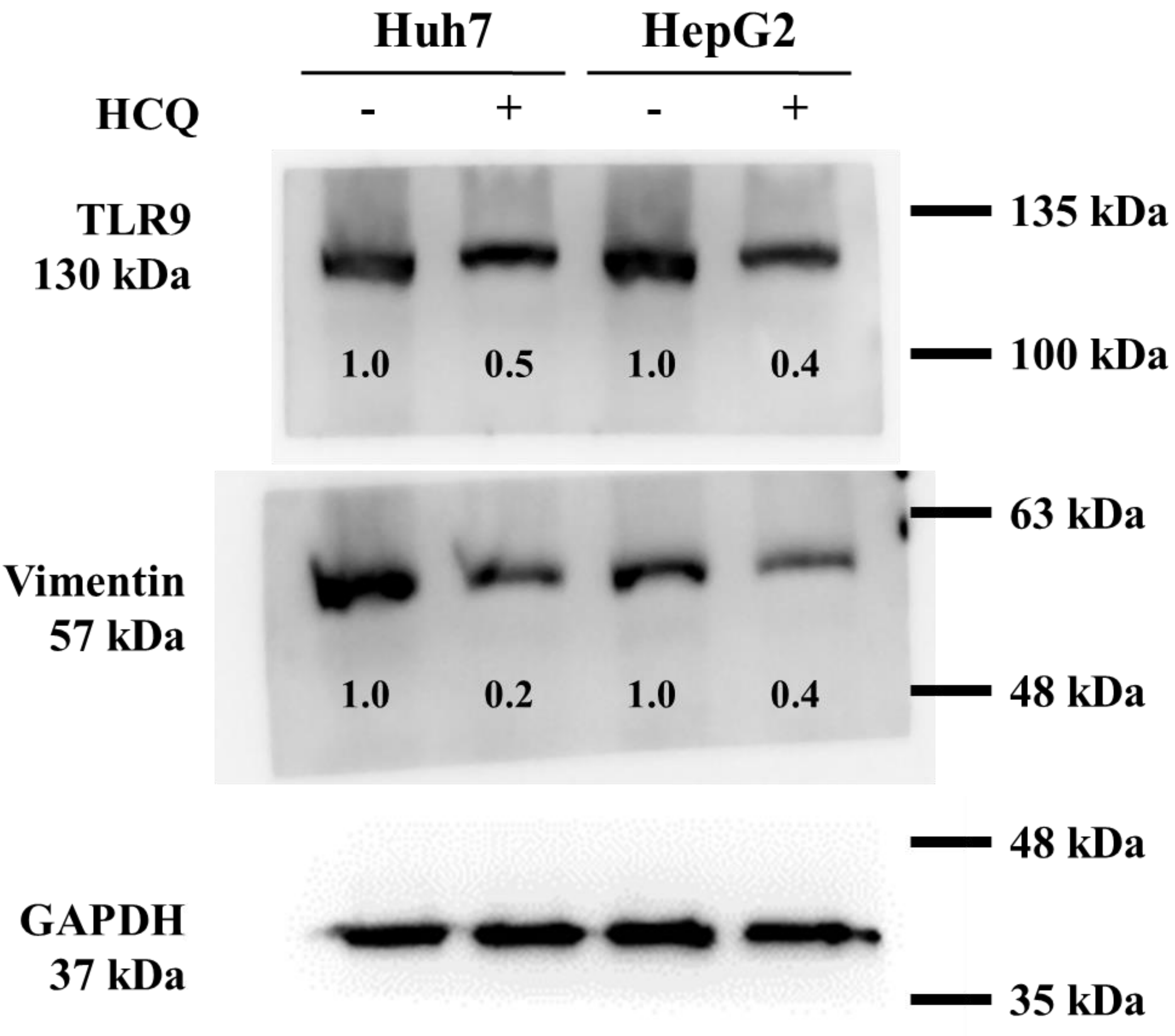

Supplementary Figure S2. Full-size blots of Figure 4D

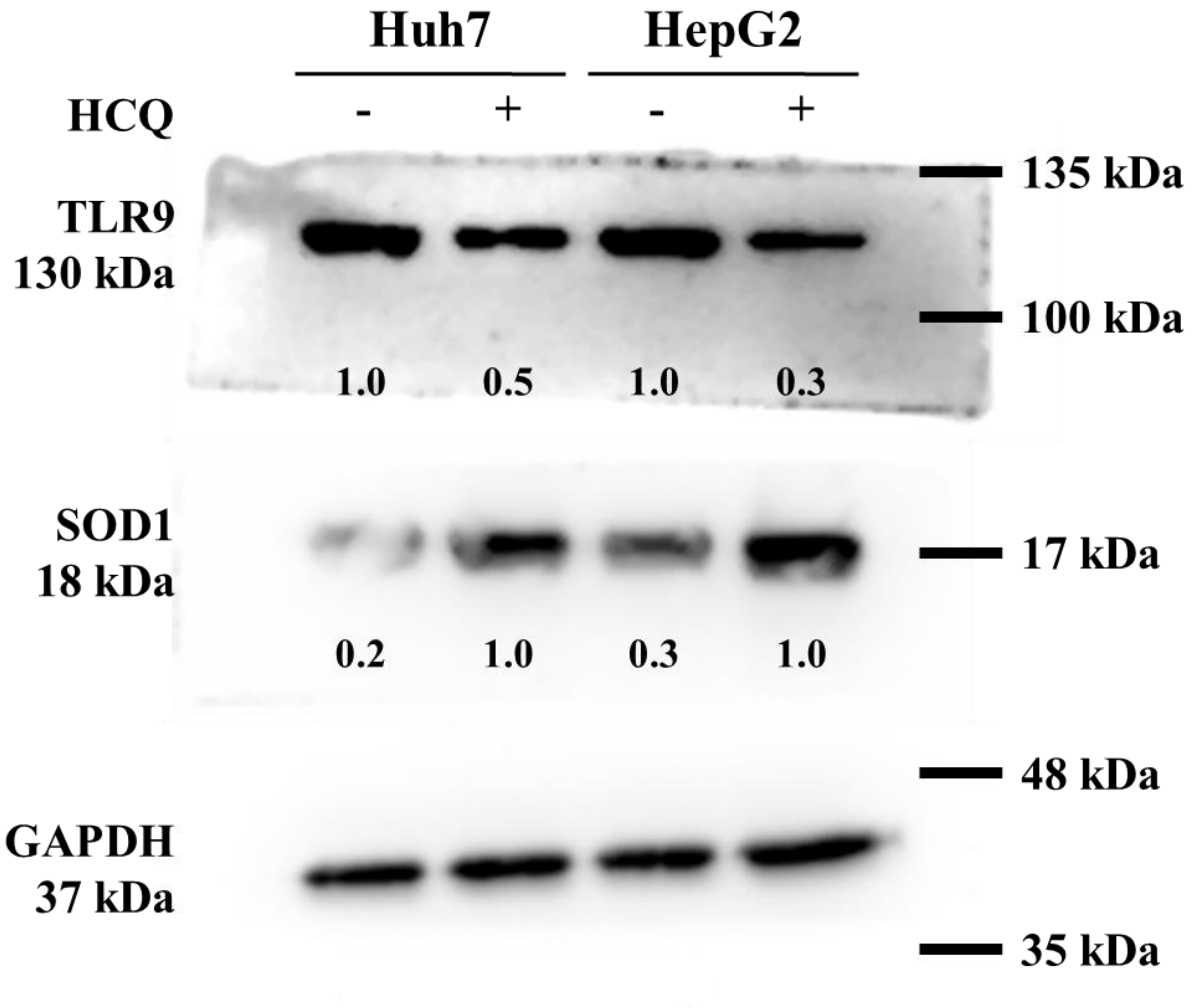

Supplementary Figure S3. Full-size blots of Figure 5D

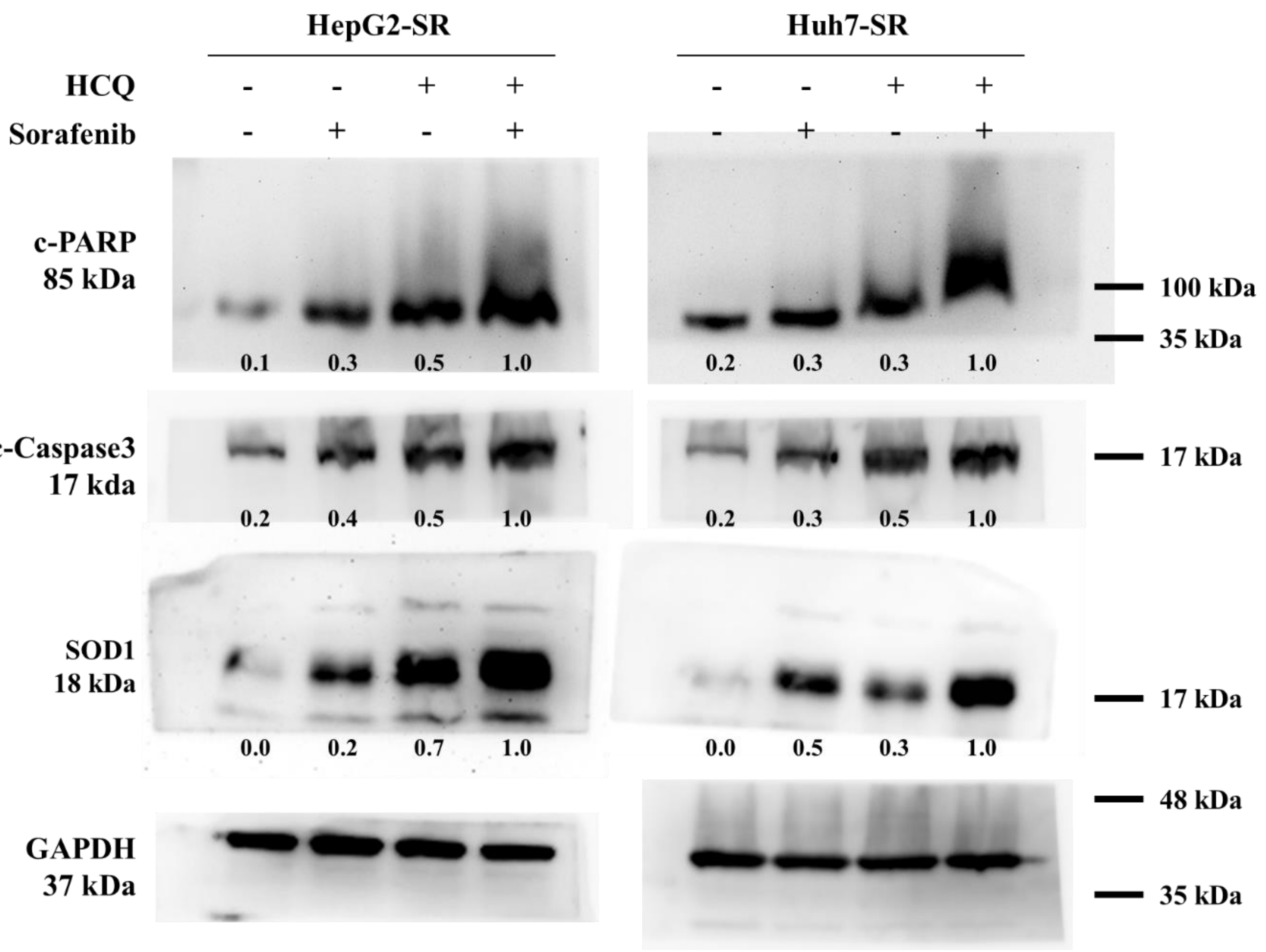

Supplementary Figure S4. Full-size blots of Figure 6D

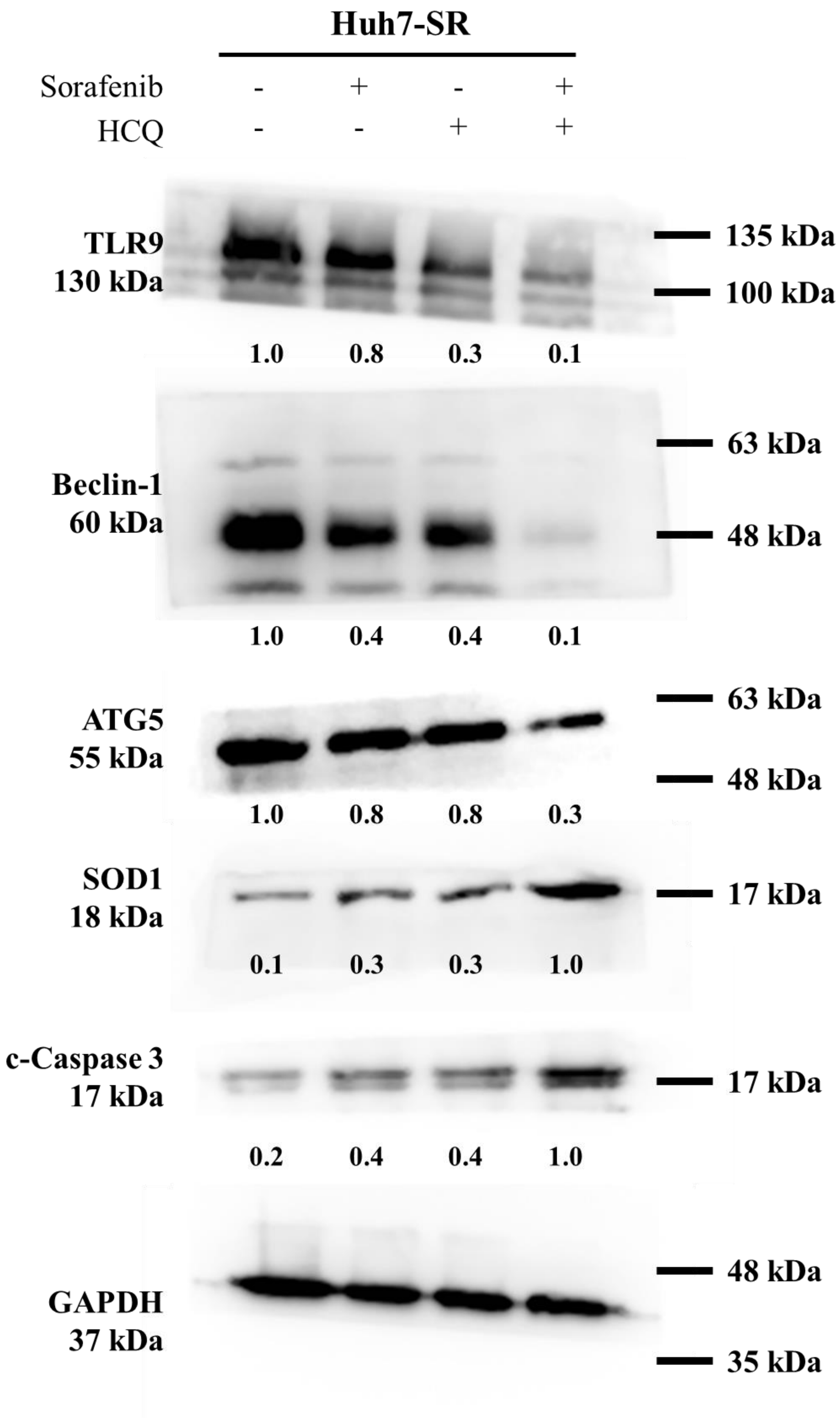

Supplementary Figure S5. Full-size blots of Figure 7E

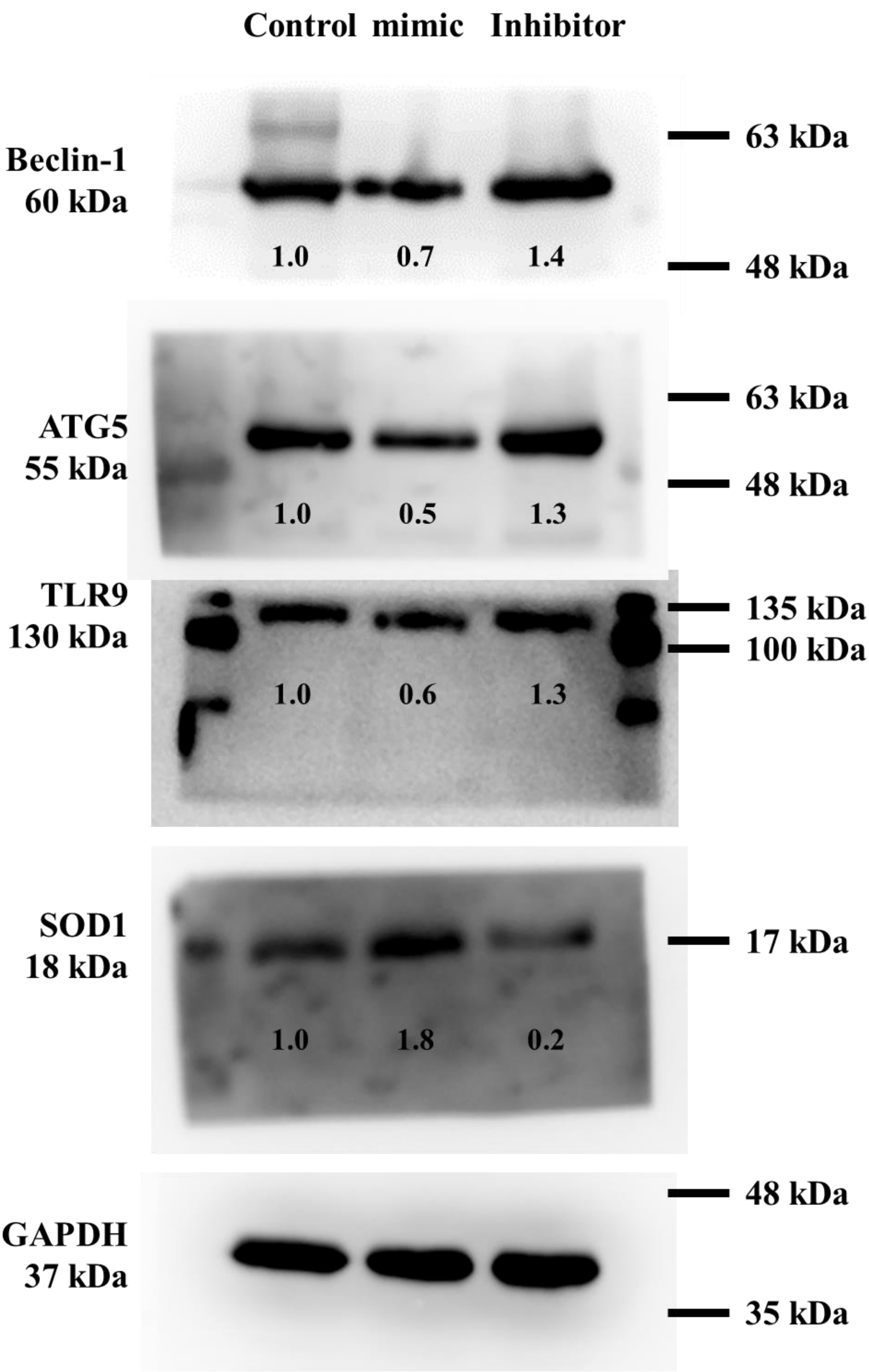

Supplementary Figure S6. Full-size blots of Figure 8F

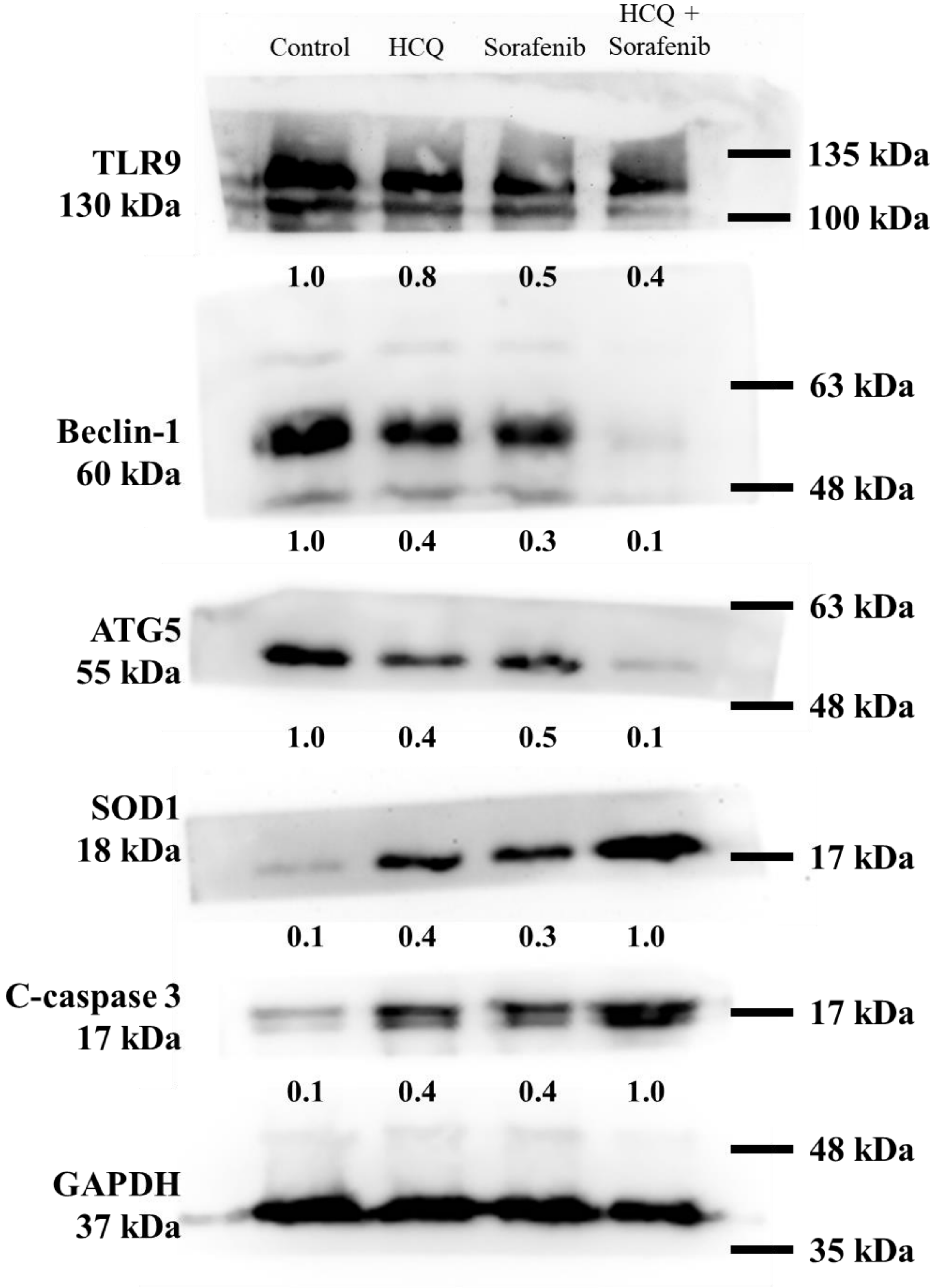

Supplementary Figure S7. Full-size blots of Figure 9D
